# Supplementary material for: Importations of SARS-CoV-2 lineages decline after nonpharmaceutical interventions in phylogeographic analyses
Source: Nat Commun. 2024 Jun 20;15:5267. doi: 10.1038/s41467-024-48641-2 (PMC11190289; doi:10.1038/s41467-024-48641-2)
Supplement: Supplementary file 3 — Reporting Summary [file 41467_2024_48641_MOESM3_ESM.pdf]

Corresponding author(s): AMC

Last updated by author(s): Apr 18, 2024

Reporting Summary

Nature Portfolio wishes to improve the reproducibility of the work that we publish. This form provides structure for consistency and transparency in reporting. For further information on Nature Portfolio policies, see our Editorial Policies and the Editorial Policy Checklist.

Statistics

For all statistical analyses, confirm that the following items are present in the figure legend, table legend, main text, or Methods section.

n/a	Confirmed	
☐	☒	The exact sample size (*n*) for each experimental group/condition, given as a discrete number and unit of measurement
☒	☐	A statement on whether measurements were taken from distinct samples or whether the same sample was measured repeatedly
☒	☐	The statistical test(s) used AND whether they are one- or two-sided<br>*Only common tests should be described solely by name; describe more complex techniques in the Methods section.*
☒	☐	A description of all covariates tested
☒	☐	A description of any assumptions or corrections, such as tests of normality and adjustment for multiple comparisons
☐	☒	A full description of the statistical parameters including central tendency (e.g. means) or other basic estimates (e.g. regression coefficient) AND variation (e.g. standard deviation) or associated estimates of uncertainty (e.g. confidence intervals)
☒	☐	For null hypothesis testing, the test statistic (e.g. *F*, *t*, *r*) with confidence intervals, effect sizes, degrees of freedom and *P* value noted<br>*Give P values as exact values whenever suitable.*
☐	☒	For Bayesian analysis, information on the choice of priors and Markov chain Monte Carlo settings
☒	☐	For hierarchical and complex designs, identification of the appropriate level for tests and full reporting of outcomes
☒	☐	Estimates of effect sizes (e.g. Cohen's *d*, Pearson's *r*), indicating how they were calculated

Our web collection on statistics for biologists contains articles on many of the points above.

Software and code

Policy information about availability of computer code

Data collection	N/A. No software is used for data collection.
Data analysis	The data were analyzed through customized codes all available at https://github.com/hzi-bifo/covid-germany-mcmc. The Bayesian analyses done with Thorney BEAST version 0.1.1 (https://beast.community/thorney_beast). For building the initial trees sarscov2phylo workflow version 22-7-20 was used (https://github.com/roblanf/sarscov2phylo).

For manuscripts utilizing custom algorithms or software that are central to the research but not yet described in published literature, software must be made available to editors and reviewers. We strongly encourage code deposition in a community repository (e.g. GitHub). See the Nature Portfolio guidelines for submitting code & software for further information.

Data

Policy information about availability of data

All manuscripts must include a data availability statement. This statement should provide the following information, where applicable:

- Accession codes, unique identifiers, or web links for publicly available datasets
- A description of any restrictions on data availability
- For clinical datasets or third party data, please ensure that the statement adheres to our policy

The dataset was downloaded from GISAID and is accessible through their website. The Accession IDs for genome sequences are available at https://github.com/hzi-bifo/covid-germany-mcmc.

Research involving human participants, their data, or biological material

Policy information about studies with human participants or human data. See also policy information about sex, gender (identity/presentation), and sexual orientation and race, ethnicity and racism.

Reporting on sex and gender	N/A
Reporting on race, ethnicity, or other socially relevant groupings	N/A
Population characteristics	N/A
Recruitment	N/A
Ethics oversight	N/A

Note that full information on the approval of the study protocol must also be provided in the manuscript.

Field-specific reporting

Please select the one below that is the best fit for your research. If you are not sure, read the appropriate sections before making your selection.

☒ Life sciences ☐ Behavioural & social sciences ☐ Ecological, evolutionary & environmental sciences

For a reference copy of the document with all sections, see nature.com/documents/nr-reporting-summary-flat.pdf

Life sciences study design

All studies must disclose on these points even when the disclosure is negative.

Sample size	We used all the SARS-CoV-2 genomic sequences available at GISAID on June 2nd, 2021.
Data exclusions	Samples with missing or ambiguous locations and erroneous dates, i.e., with sampling dates earlier than the first confirmed occurrence of the respective lineage, were removed. Low-quality samples, corresponding to sequences shorter than 28,000 bp or with more than 1000 ambiguous bases, were removed as well.
Replication	The accession id of the GISAID sequences are available, after obtaining the metadata and sequences files from the GISAID database, while all the codes are available at the GitHub repository (https://github.com/hzi-bifo/covid-germany-mcmc), all the results could be reproduced with the available codes.
Randomization	Following these approaches, we adapted a subsampling approach to ensure computational tractability. The data set was stratified based on the Pango lineage assignments. Specifically, we identified twelve Pango lineages with more than 1000 sequences overall and more than 40 from Germany: A, B.1.1.7, B.1.1.519, B.1.1.70, B.1.1.317, B.1.177, B.1.160, B.1.221, B.1.36, B.1.258, B.1.351, and C. Of the sequences assigned to these Pango lineages, we removed sequences with dates not in the valid format or containing incomplete information (only year or year and month) or with dates prior to the first observed case of their respective Pango lineage, as in (Rambaut et al. 2020). The number of samples of the selected Pango-lineages, after filtering out invalid dates, is 1,067,284 out of 1,729,077 (61.7%). The number of samples from the selected Pango lineages within Germany is 108,089 out of 119,801 (90.2%). Furthermore, the analyses are conducted independently on samples from each Pango-lineage. Consequently, the inferred lineages are entirely contained within one of the selected Pango-lineages. We relied on the Pango lineage assignment provided by GISAID in the metadata file. This process resulted in a sequence set eligible for subsampling, covering the time frame from December 30, 2019, to May 31, 2021.<br>Similar to the studies of Bbosa et al., Bollen et al., Hodcroft et al., Lemey et al., and Nemira et al., [5–9] we subsampled viral genome sequences using sampling dates, confirmed case numbers, and the country of origin, to account for varying data availability. Specifically, we set the maximum number of total samples per week to 10,000 across all countries, and calculated the maximum sample size per country by distributing this value (10,000) proportionally to the number of confirmed cases for the countries. For each country, we then either sampled the specified number of sequences or all available sequences, if the number was lower. To ensure that the main lineages were well sampled, we furthermore added the five earliest and latest samples for each lineage to the data set. The final dataset includes 1194 samples from Germany and 16,486 from other countries.<br>To assess the consistency of results across sampling schemes, we created two more datasets using different genome subsampling schemes for phylogeographic analyses [6,10]. First, we randomly subsampled equal numbers of up to 100 non-German sequences per week from all countries combined together with 100 German sequences, depending on availability. Second, we randomly sampled up to 25 sequences per week from the genome dataset for each country other than Germany, and 100 sequences per week for Germany. To ensure the main lineages were sampled, we then added the five earliest and latest samples for each, as before, and randomly sampled additional sequences to reach at least 1000 sequences for each lineage per dataset. These additional datasets include 2,689 and 2,691 sequences from Germany and 1,932 and 25,107 non-German sequences in the 50:50 and 25:100 datasets, respectively.
Blinding	Since we used publicly available dataset, the authors were blind to any information regarding the samples, except the location and sampling dates.

Reporting for specific materials, systems and methods

We require information from authors about some types of materials, experimental systems and methods used in many studies. Here, indicate whether each material, system or method listed is relevant to your study. If you are not sure if a list item applies to your research, read the appropriate section before selecting a response.

Materials & experimental systems

n/a	Involved in the study
☒	☐ Antibodies
☒	☐ Eukaryotic cell lines
☒	☐ Palaeontology and archaeology
☒	☐ Animals and other organisms
☒	☐ Clinical data
☒	☐ Dual use research of concern
☒	☐ Plants

Methods

n/a	Involved in the study
☒	☐ ChIP-seq
☒	☐ Flow cytometry
☒	☐ MRI-based neuroimaging

Plants

Seed stocks	*Report on the source of all seed stocks or other plant material used. If applicable, state the seed stock centre and catalogue number. If plant specimens were collected from the field, describe the collection location, date and sampling procedures.*
Novel plant genotypes	*Describe the methods by which all novel plant genotypes were produced. This includes those generated by transgenic approaches, gene editing, chemical/radiation-based mutagenesis and hybridization. For transgenic lines, describe the transformation method, the number of independent lines analyzed and the generation upon which experiments were performed. For gene-edited lines, describe the editor used, the endogenous sequence targeted for editing, the targeting guide RNA sequence (if applicable) and how the editor was applied.*
Authentication	*Describe any authentication procedures for each seed stock used or novel genotype generated. Describe any experiments used to assess the effect of a mutation and, where applicable, how potential secondary effects (e.g. second site T-DNA insertions, mosiacism, off-target gene editing) were examined.*
